# A hypothesis about interrelations of epigenetic factors and transposable elements in memory formation

**DOI:** 10.18699/vjgb-24-54

**Published:** 2024-09

**Authors:** R.N. Mustafin

**Affiliations:** Bashkir State Medical University, Ufa, Russia

**Keywords:** long noncoding RNAs, long-term memory, miRNAs, retroelements, transposons, epigenetic factors, длинные некодирующие РНК, долговременная память, микроРНК, ретроэлементы, транспозоны, эпигенетические факторы

## Abstract

The review describes the hypothesis that the drivers of epigenetic regulation in memory formation are transposable elements that influence the expression of specific genes in the brain. The hypothesis is confirmed by research into transposon activation in neuronal stem cells during neuronal differentiation. These changes occur in the hippocampus dentate gyrus, where a pronounced activity of transposons and their insertion near neuron-specific genes have been detected. In experiments on changing the activity of histone acetyltransferase and inhibition of DNA methyltransferase and reverse transcriptase, the involvement of epigenetic factors and retroelements in the mechanisms of memory formation has been shown. Also, a number of studies on different animals have revealed the preservation of long-term memory without the participation of synaptic plasticity. The data obtained suggest that transposons, which are genome sensors highly sensitive to various environmental and internal influences, form memory at the nuclear coding level. Therefore, long-term memory is preserved after elimination of synaptic connections. This is confirmed by the fact that the proteins involved in memory formation, including the transfer of genetic information through synapses between neurons (Arc protein), originate from transposons. Long non-coding RNAs and microRNAs also originate from transposons; their role in memory consolidation has been described. Pathological activation of transposable elements is a likely cause of neurodegenerative diseases with memory impairment. Analysis of the scientific literature allowed us to identify changes in the expression of 40 microRNAs derived from transposons in Alzheimer’s disease. For 24 of these microRNAs, the mechanisms of regulation of genes involved in the functioning of the brain have been described. It has been suggested that the microRNAs we identified could become potential tools for regulating transposon activity in the brain in order to improve memory.

## Abstract (ru)

В обзорной статье описана гипотеза, согласно которой драйверами эпигенетической регуляции в формировании памяти являются мобильные генетические элементы, влияющие на экспрессию специфических генов в головном мозге. В подтверждение приведены результаты научных исследований о закономерной активации транспозонов в нейрональных стволовых клетках при дифференцировке нейронов. Данные процессы происходят в зоне нейрогенеза – зубчатой извилине гиппокампа, где определяются наибольшая активность мобильных генетических элементов и их инсерции в локусы вблизи генов, экспрессируемых нейронами с их активацией. В экспериментах по изменению активности ацетилтрансферазы гистонов, ингибированию ДНК-метилтрансферазы и обратной транскриптазы было показано вовлечение эпигенетических факторов и ретроэлементов в механизмы формирования памяти. В то же время в ряде работ на разных животных продемонстрировано сохранение долговременной памяти без участия синаптической пластичности. Полученные данные позволяют предположить, что транспозоны, являющиеся высокочувствительными сенсорами генома к различным средовым и внутренним воздействиям, формируют память на уровне ядерного кодирования. Это отражается в изменении синаптической пластичности, чем можно объяснить сохранение долговременной памяти после устранения синаптических связей у животных. Подтверждением служат факты происхождения от мобильных генетических элементов белков, непосредственно участвующих в формировании памяти, в том числе в передаче генетической информации через синапсы между нейронами (белок Arc). Транспозоны – источники длинных некодирующих РНК и микроРНК, роль которых в консолидации памяти описана. Патологическая активация мобильных генетических элементов является вероятной причиной нейродегенеративных болезней с нарушением памяти. Анализ научной литературы позволил нам обнаружить данные об изменениях экспрессии 40 микроРНК, произошедших от транспозонов, при болезни Альцгеймера. Для 24 из этих микроРНК описаны механизмы регуляции генов, участвующих в функционировании головного мозга. Сделано предположение, что установленные нами микроРНК могли бы стать потенциальными инструментами для регуляции активности транспозонов в головном мозге с целью улучшения памяти.

## Introduction

Memory is defined as the storage and use of received information in the brain during adaptation to the environment during the life of the organism. Memory includes the processes of encoding, consolidation, storage of information, and recollection. The molecular and cellular mechanisms of memory formation are logically interpreted by the transmission of nerve impulses between the synapses of many neurons. Most modern researchers explain the processes of memory formation by synaptic plasticity (SP) – the possibility of differential changes in the strength of neuronal transmission through certain synapses (with the weakening of some and strengthening of other connections between neurons) (Ortegade San Luis, Ryan, 2022). There are four levels of memory study: psychological, neurophysiological, biochemical and cybernetic. According to the neurophysiological concept, memory is divided into short-term and long-term memory (LTM). Most modern neurophysiological theories boil down to the role of synaptic plasticity in the formation of LTM, which is closely related to the biochemical theory, since the electrochemical reaction of the formation of a nerve impulse turns into the biochemical process of the formation of new proteins (Munin, Olenko, 2022).

Reconsideration of the classical hypothesis of synaptic plasticity is necessary in connection with the growing evidence of memory retention without the participation of synaptic plasticity. The results of the first experiments in this area were published in 1984. Preservation of odor avoidance memory formed at the caterpillar stage was revealed in mature Manduca sexta moths after metamorphosis with reorganization of synapses (Levine, 1984). The memory of recognizing a textured surface to determine the presence of food was retained in planarians after head removal and subsequent brain regeneration (Shomrat, Levin, 2013). In a coculture of motor and sensory neurons from the sea hare Aplysia, memory for training with interval serotonin pulses persisted after its apparent elimination by anti-mnemonic drugs that erase learning-associated synaptic growth (Chen et al., 2014). In experiments on mice, the restoration of fear memory was determined when engram cells were reactivated in the absence of synaptic changes (after administration of the protein synthesis inhibitor anisomycin) (Ryan T.J. et al., 2015).

Various genes are involved in the formation of LTM, the most famous of which is CREB (cAMP-responsive element binding protein). Mutations in the CREB gene cause memory deficits in mice (Hegde, Smith, 2019). The CREB gene product, together with glucocorticoid receptors, is involved in the intracellular mechanisms of the influence of glucocorticoids on LTM formation in the hippocampus (Buurstede et al., 2022).

Experiments on Drosophila demonstrated the role of the beta-catenin gene (CTNNB1) in the consolidation of LTM due to its effect on Wnt signaling pathways (Tan Y. et al., 2013). Systematic reviews of data accumulated in the scientific literature have shown a stimulating effect of transcription factors on memory development, which are protein products of the expression of the following genes: NF-κB (Kaltschmidt B., Kaltschmidt C., 2015), Zif268, XBP1, Srf, Npas4, Foxp1, Crtc1, c-Rel (Hegde, Smith, 2019). In addition to the genes necessary for memory consolidation, which also include NR2B (encodes a subunit of the inotropic glutamate receptor Nmethyl- d-aspartate), memory suppressor genes, which include AIM2, ATF4, BChE, Bec1, CCR5, Cdk5, Crtl1, Diap1, Dicer1, DFF45, GABAaB3, GABAARα4, Gabra 4, Galectin-3, GAT1, QR2, Np65, Hcn1, Hdac2, Mef2, Kvβ1.1, PDE1b, Paip2a, Pkr, GCN2, IRS2, RGS14, RARalpha, p75NTR, PDE4A, Ogg1, PERK, RPTPsigma, Piwi1, Piwi2, S100b, TLCN, Pde4d/8b, 11b-HSD1, are important in the regulation of LTM (Noyes et al., 2021).

The results obtained indicate the presence of other mechanisms for maintaining LTM, which are realized in the form of synaptic plasticity. Most likely, memory is consolidated at the level of nuclear DNA under the driver influence of transposable elements (TEs), which rearrange the chromatin structure upon their activation, and are also integrated into specific loci during neuronal differentiation (Perrat et al., 2013; Upton et al., 2015). Chromatin remodeling under the influence of epigenetic modifications is necessary for the preservation and maintenance of memory, since it is reflected in changes in gene regulation in the brain. Epigenetic factors include cytosine methylation at CpG dinucleotides, chromatin modifications, and non-coding RNAs (ncRNAs), all of which are involved in long-term memory formation (Lipsky, 2013). The role of epigenetic factors in memory formation has been proven in experiments. Exposure to a DNA methyltransferase inhibitor destroyed fully consolidated fear memory one month after its onset (Miller et al., 2010).

Enhancing histone acetylation by manipulating the activity of a specific isoform of histone acetyltransferase (HAT) in neurons significantly reduced memory consolidation (Jarome, Lubin, 2014). The formation of LTM is influenced by specific histone modifications: H2BK120ub, H3K9me2, H3K36me3, H3K27me3, H3K9me3, H3K4me3, H3K14ac, H3K9ac (Hegde, Smith, 2019).

At the same time, DNA methylation and chromatin reorganization enzymes interact with microRNAs (Mustafin, Khusnutdinova, 2017), which can also serve as guides recognizing complementary genome sequences in the mechanism of RNAdependent DNA methylation (Chalertpet et al., 2019). This phenomenon suggests the role of TEs as drivers of epigenetic regulation of memory, since transposons are important sources for the emergence of microRNA genes (Wei et al., 2016). The role for TEs in regulation of neuronal function in humans was suggested in a 2022 review (Chesnokova et al., 2022). Although TEs cannot be the drivers of all epigenetic changes associated with mnestic processes, they are the evolutionary sources of many microRNAs (Wei et al., 2016), most long non-coding RNAs (lncRNAs) (Johnson, Guigo, 2014), and can themselves be transcribed directly into lncRNAs (Lu X. et al., 2014; Honson, Macfarlan, 2018). Consequently, transposons, to one degree or another, participate in most epigenetic and gene networks regulating genome functioning. In addition, TEs themselves are under the control of epigenetic changes, in part due to the mutual regulatory effects of microRNAs derived from them (Mustafin, Khusnutdinova, 2017).

Epigenetic regulation of transposons involves KRAB zinc finger proteins via heterochromatin initiation complexes, which modify histones and alter DNA methylation (Wolf et al., 2015). More than half of the binding regions for the PLZF (Promyelocytic Leukemia Zinc Finger) protein, a member of the Krüppel-type zinc finger family, are located within the LINE1 element genes (Lapp, Hunter, 2016). The SOX2 and HDAC1 control LINE1 activity by binding to the transcriptional repressor methyl-CpG-linked protein-2 (MeCP2). There are many other ways to regulate transposon activity, which include the APOBEC3 (Mager, Stoye, 2014), APOBEC1, ERCC, TREX1, RB1, HELLS, MEGP2 (Rodic, Burns, 2013), SIRT6 proteins (Van Meter et al., 2014).

In the MDTE DB database, 661 human microRNAs derived from TEs have been published (Wei et al., 2016). In neuronal stem cells (NSCs), transposon activation promotes genomic mosaicism of maturing neurons, which is necessary for their differentiation (Muotri et al., 2005). These changes are found in the zone of neurogenesis, the dentate gyrus of the hippocampus, not only in experimental animals, but also in humans (Coufal et al., 2009; Baillie et al., 2011; Kurnosov et al., 2015). In this case, TEs are inserted into genes or near genes involved in the functioning of neurons (Upton et al., 2015), and the hippocampus plays a key role in learning and memory formation (Zhang H. et al., 2021).

TEs can be activated under the influence of environmental factors, the signals of which enter the brain through neural networks, since transposons are highly sensitive sensors of environmental and internal changes (Mustafin, Khusnutdinova, 2019). Transposons are regions of the genome that move within the genome using the mechanism of “cut and paste” (DNA transposons) and “copy and paste” (retroelements – RE). REs may contain long terminal repeats (LTRREs) or not contain them (non-LTR-REs). In humans, the latter include autonomous REs (LINEs – long interspersed nuclear elements) and non-autonomous ones (SINEs – short interspersed nuclear elements, SVA – SINE-VNTR-Alu elements) (Mustafin, Khusnutdinova, 2017).

Most lncRNAs, like microRNAs, are derived from transposons. On average, 41 % of lncRNA exons contain RE sequences, and 83 % of them contain at least one transposon fragment (Johnson, Guigo, 2014; Wei et al., 2016). Moreover, LINE1 transcripts themselves function as lncRNAs, interacting with specific regions of chromatin and regulating gene expression (Honson, Macfarlan, 2018), and LTR-REs serve as genes for many lncRNAs (Lu X. et al., 2014). Therefore, the participation of ncRNAs in memory storage indicates the importance of transposons in these processes.

## The role of non-coding RNAs in memory formation

The tissue specificity of lncRNAs exceeds that of proteins. In the regulation of stem cell differentiation, they interact with REs (Ramsay et al., 2017). lncRNAs are formed from intergenic regions of eukaryotic genomes, characterized by tissue-specific transcription, from overlapping and antisense patterns relative to adjacent genes, which they regulate (Arendt et al., 2017). This allows them to determine a variety of cellular phenotypes, especially in the brain (Lapp, Hunter, 2016), which may reflect the role of transposons in these processes (Coufal et al., 2009; Baillie et al., 2011). RNA sequencing analysis with induction of long-term potentiation (LTP) in the dentate gyrus of rats after high-frequency stimulation of the perforant pathway showed a positive, pronounced correlation of the dynamic expression of lncRNAs with REs and proteincoding genes (Maag et al., 2015).

A number of scientific works have shown the role of specific lncRNAs in memory consolidation. Experiments on rodents revealed that lncRNA NEAT1 is an epigenetic suppressor of LTM formation in the hippocampus (Butler et al., 2019). Increased expression of the lncRNA SLAMR was detected in hippocampal neurons under the influence of contextually conditioned fear. SLAMR is transported to dendrites via the molecular motor KIF5C and is recruited to the synapse in response to stimulation. SLAMR modulates the activity of the CaMKIIα protein, which plays an important role in synaptic plasticity in synaptoneurosomes (Espadas et al., 2023). lncRNA Carip also interacts with the CaMKII protein, which controls the phosphorylation of AMPA and NMDA receptors in the hippocampus, affecting spatial memory. In the absence of Carip, synaptic plasticity dysfunction occurs in CA3-CA1 in the hippocampus, indicating the role of this lncRNA in memory regulation (Cui et al., 2022). Since many lncRNAs are expressed in the brain, they may regulate genes of LTM (Samaddar, Bnerjee, 2021).

At least 70 % of human miRNAs are expressed in the brain, with a specific miRNA activation pattern for each region (Chen, Qin, 2015). In hippocampal neurons, induction 

## The role of retroelements in the consolidation of long-term memory

The role of REs in the formation of long-term memory is evidenced by the results of experimental work of independent researchers. Thus, by inhibiting LINE1 in the hippocampus of mice, the role of REs in memory consolidation resulting from genomic mosaicism was determined. To do this, the animals were placed on an illuminated side, after which they were allowed to move to the dark side of the chamber, where they were exposed to current. The learning memory was reflected in an increase in mouse latency when moving to the dark side of the chamber. Long-term memory was significantly impaired 72 hours after hippocampal administration of the reverse transcriptase inhibitor lamivudine (Bachiller et al., 2017). Another study of context-dependent fear memory, in addition to demonstrating significant suppression of longterm memory following lamivudine administration, identified LINE1 expression in the hippocampus and prefrontal cortex during fear memory using quantitative real-time PCR of hippocampal and prefrontal cortex samples (Zhang W.J. et al., 2021). A significant number of TEs transpositions (more than 200 per cell) in memory-related neurons have also been identified in the Drosophila brain (Perrat et al., 2013). Since the results of many of the studies cited in the review reflect correlational relationships and require more direct confirmation of the effect of transposons on memory, such as singlecell sequencing of the hippocampal region and specific areas of the cerebral cortex before and after long-term memory training.

According to data from the ENCODE and FANTOM consortia, transposon activity depends on the cell type and affects the expression of neighboring genes. TEs are of greatest importance in brain function regulation, providing adaptive functions of the central nervous system. In response to the effects of steroids, epigenetic and environmental factors, they change the functioning of neurotransmitter systems to adapt to changing environmental conditions, including LTM formation (Lapp, Hunter, 2016). Activated REs play a regulatory role not only for NSCs, but also in the late phase of neuronal differentiation (Muotri et al., 2010), controlling the specific pattern of gene expression in neurons located in certain areas of the brain, due to which memory is formed (Singer et al., 2010). In the mouse hippocampus, SINE expression profiles are cell type specific. In response to brief exposure to a novel stimulus, SINEs are activated in dentate granule neurons over a time course similar to that of protein-coding genes (Linker et al., 2020).

LTR-REs play an important role in the development of long-term memory. The protein product of the HERV env gene activates BDNF (brain-derived neurotrophic factor) (Huang et al., 2011), which is involved in synaptic transmission and LTP in the hippocampus and other brain regions for the formation of various forms of memory (Leal et al., 2014). In evolution, the domestication of LTR-REs led to the formation of genes that are key to long-term memory. According to computer analysis (Campillos et al., 2006), confirmed by phylogenetic studies (Ashley et al., 2018; Pastuzyn et al., 2018), the Arc gene (Activity-regulated cytoskeleton-associated protein) in humans originated from ERV Ty3/gypsy. The Arc protein regulates synaptic plasticity in the control of signaling networks during memory consolidation. Arc gene transcripts are transported to dendrite synapses, where they are synthesized into protein on ribosomes. Arc forms a capsid that encapsulates its own mRNAs. The resulting virus-like structures are loaded into extracellular vesicles and transported to neurons, transmitting genetic information and regulatory signals through neural networks, which is necessary for the formation of LTM (Ashley et al., 2018; Pastuzyn et al., 2018).

The Prp8 protein, which is a component of the eukaryotic spliceosome, evolved from ERV reverse transcriptase (Dlakić, Mushegian, 2011). Experiments on Drosophila demonstrated the key role of Prp8 in controlling the expression of the neuropeptide FMRFa in neurons (Cobeta et al., 2018). The TERT protein, derived from retroelement reverse transcriptase (Kopera et al., 2011), regulates spatial memory formation by modulating neuronal development in the hippocampus (Zhou et al., 2017). The Gag ERV protein gave rise to the PEG10 protein, which interacts with ATXN2 and ATXN10 in stress granules and extracellular vesicles and regulates neuronal mi-gration during LTM formation (Pandya et al., 2021). In evolution, Gag also became the source of the CCHC type of zinc finger protein (encoded by the SIRH11/ZCCHC16 gene). Deletion of the SIRH11/ZCCHC16 gene in mice causes abnormal behavior associated with cognitive abilities, including working memory (Kaneko-Ishino, Ishino, 2016). The Gag gene was the origin of the RTL1/PEG11 gene expressed in the brain. Mice with knockout of the paternal allele (Rtl1m+/p–) showed decreased memory (Chou et al., 2022). The data obtained on the role of ERV-derived proteins in LTM formation indicate the potential participation of ERVs themselves in these processes.

Thus, the strength of the hypothesis of the role of transposons in the formation of long-term memory is the presence of direct experimental evidence of the participation of REs in these processes (Singer et al., 2010; Huang et al., 2011; Perrat et al., 2013; Leal et al., 2014; Lapp, Hunter, 2016; Bachiller et al., 2017; Zhang W.J. et al., 2021). There is also indirect evidence of the importance of mobile genetic elements in the mechanisms of long-term memory, since REs are the evolutionary sources of proteins involved in the formation of memory, such as Arc (Campillos et al., 2006; Ashley et al., 2018; Pastuzyn et al., 2018), Prp8 (Dlakić, Mushegian, 2011; Cobeta et al., 2018), TERT (Kopera et al., 2011; Zhou et al., 2017), PEG10 (Pandya et al., 2021), CCHC type zinc finger protein (Kaneko-Ishino, Ishino, 2016).

TEs are also sources of microRNAs (Wei et al., 2016) and long ncRNAs (Johnson, Guigo, 2014), which are actively involved in the epigenetic regulation of LTM. Therefore, the strength of the proposed hypothesis is the explanation of the mechanisms of influence of environmental stimuli on epigenetic factors, since in these processes REs are effective mediators, sensitive not only to stress, but also to many external and internal factors, perceiving information for the adaptation of the body (Mustafin, Khusnutdinova, 2019), which corresponds to the definition of memory (Ortega-de San Luis, Ryan, 2022). Moreover, the ability of REs to insert into new loci of the genome, thereby changing the expression of specific genes involved in the formation of long-term memory, in contrast to the synaptic plasticity hypothesis, explains long-term consolidation at the genome level (Perrat et al., 2013; Bachiller et al., 2017; Zhang W.J. et al., 2021).

The proposed hypothesis is also supported by the high rate of information consolidation at the genome level (compared to the possible influence of the potential on protein synthesis on ribosomes) due to the activation of REs, since TEs are involved in many gene and epigenetic networks (due to the presence of sequences complementary to non-coding RNAs derived from TEs). Therefore, environmental stimuli that activate TEs can quickly affect changes in gene networks, which is sufficient for the formation of LTM.

A possible counterargument to the role of transposons in memory formation can be the fact of activation of REs during aging, which is characterized by a decline in cognitive functions and memory. However, a systematic review of the scientific literature showed that the cause of aging is a pathological activation of REs (Mustafin, Khusnutdinova, 2018a), while for ontogenesis, starting from the division of the zygote and until reaching the sexually mature state of the organism, species-specific activation of strictly defined transposons occurs, including in the brain (Mustafin, Khusnutdinova, 2018b). This statement is supported by pathological activation of REs in diseases associated with old age, characterized by impaired long-term memory.

## Relationship between transposons and microRNAs in memory disorders

Prospects for studying the relationship of TEs with epigenetic factors in the formation of LTM in health and disease are associated with the possibility of correcting disorders, since epigenetic changes are reversible. Although TE expression is required for normal memory consolidation, their pathological activation is a factor in the development of neurodegenerative diseases. Experiments on mice modeled for AD showed impairment of long-term memory due to derepression of REs (El Hajjar et al., 2019). G-quadruplex derived from evolutionarily conserved LINE1 suppresses gene expression in Alzheimer’s disease neurons (Hanna et al., 2021). In the mouse brain, tau proteins activate ERVs, increasing their DNA copy numbers (Ramirez et al., 2022), and in patients with Alzheimer’s disease, decondensation of heterochromatin and reduction in piwi and piRNA levels activate HERVs (Sun W. et al., 2018), LINE1 and Alu (Grundman et al., 2021).

Since microRNAs are also characterized by changes in expression during normal memory formation (Leal et al., 2014; Chen, Qin, 2015; Michely et al., 2017; Grinkevich, 2020) and in Alzheimer’s disease (Patel et al., 2019), these changes may be associated with pathological TE activation. To confirm this assumption, we analyzed the scientific literature on the relationship between microRNAs and TEs in Alzheimer’s disease. For this purpose, we studied the results of studies on changes in the expression of microRNAs derived from mobile genetic elements (according to the MDTE database (Wei et al., 2016)) in Alzheimer’s disease. As a result, 40 such microRNAs were discovered, which indicate the role of microRNAs interconnected (due to complementarity of nucleotide sequences) with TEs in the mechanisms of memory formation in humans under normal and pathological conditions. For 24 of these miRNAs, functional significance in the brain was described (see the Table).

**Table 1.**
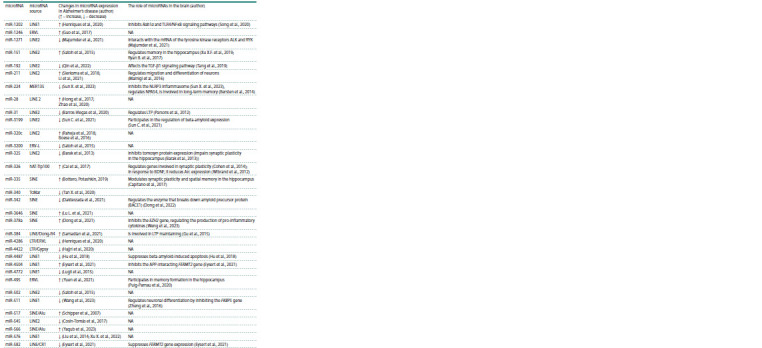
Association of transposon-derived microRNAs with Alzheimer’s disease Note. NA – no data available.

**Table 1(end).**
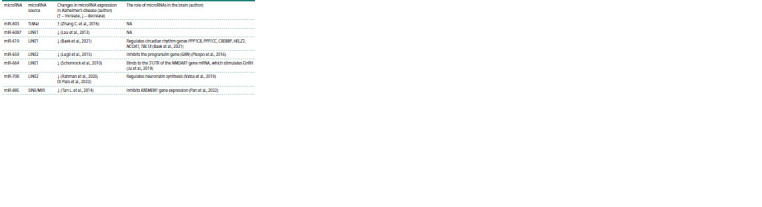
Association of transposon-derived microRNAs with Alzheimer’s disease Note. NA – no data available.

## Conclusion

Investigation of the role of epigenetic factors in normal and pathological long-term memory formation is promising due to the reversibility of changes occurring under their influence and the possibility of influencing them with the help of microRNAs. The most likely drivers of epigenetic regulation of genes during memory formation are TEs – highly sensitive genome sensors to environmental and internal influences. This is evidenced by the preservation of long-term memory with the complete elimination of synaptic connections. TEs consolidate memory at the level of nuclear DNA due to a programmed pattern of their activation and transposition. An analysis of the scientific literature made it possible to find evidence of the role of TEs, lncRNAs and microRNAs interconnected with them in the formation of memory in health and disease. In Alzheimer’s disease, changes in the expression of 40 microRNAs derived from TEs were determined, the majority of which originate from REs (24 microRNAs – from LINEs, 7 – from SINEs, 5 – from ERVs).

It can be assumed that in the future the identified microRNAs may become objects and tools for regulating the activity of TEs in the brain. The proposed hypothesis of the role of REs in the formation of LTM explains the missing links in the theory of synaptic plasticity, since activated transposons form insertions in specific genomic loci that change the expression of genes involved in the development of memory, which explains the consolidation of LTM at the level of nuclear coding.

## Conflict of interest

The authors declare no conflict of interest.
